# The role of agitated saline contrast echocardiography in the evaluation of pulmonary hypertension

**DOI:** 10.47487/apcyccv.v6i2.486

**Published:** 2025-06-27

**Authors:** Emilio Herrera, Jhon F. Salamanca, Luisa F. Durango

**Affiliations:** 1 Universidad Pontificia Bolivariana, Medellín, Colombia. Universidad Pontificia Bolivariana Universidad Pontificia Bolivariana Medellín Colombia; 2 Unidad Cardiovascular, Servicio de Cardiología Clínica, Clínica Medellín, Medellín, Colombia. Unidad Cardiovascular Servicio de Cardiología Clínica Clínica Medellín Medellín Colombia; 3 Departamento de Ecocardiografía, Ayudas Diagnósticas Cardiovasculares, Clínica Cardio VID, Medellín, Colombia. Departamento de Ecocardiografía Ayudas Diagnósticas Cardiovasculares Clínica Cardio VID Medellín Colombia; 4 Clínica CardioVID. Medellín, Colombia Clínica CardioVID Medellín Colombia

**Keywords:** Pulmonary Hypertension, Echocardiography, Contrast Echocardiography, Heart Septal Defects, Atrial, Hipertensión Pulmonar, Ecocardiografía, Ecocardiografía de Contraste, Defectos del Tabique Interatrial

## Abstract

The diagnostic approach to pulmonary hypertension using cardiac imaging, particularly echocardiography, provides a practical, accessible, and highly valuable tool. It helps establish the initial diagnostic probability, offers prognostic information, and supports aetiological assessment. The agitated saline contrast test, also referred to as bubble contrast echocardiography, can aid not only in confirming the diagnosis but also in characterising the condition and identifying various underlying causes of pulmonary hypertension.

## RESUMEN

El abordaje diagnóstico de la hipertensión pulmonar mediante imágenes cardíacas, como la ecocardiografía, representa una ayuda práctica, accesible y una herramienta muy valiosa; define la probabilidad inicial diagnostica; además, brinda parámetros pronósticos y puede ayudar en el diagnóstico etiológico. La prueba de solución salina agitada, contraste con solución salina/burbujas o prueba de burbujas por ecocardiografía puede orientar no solo en el diagnóstico, sino también en la caracterización e identificación de distintas causas de hipertensión pulmonar.

## Introduction

Pulmonary hypertension (PH) is a common condition that affects individuals of all ages, with an estimated prevalence of approximately 1% in the general population. Due to the higher incidence of cardiac and pulmonary diseases, it is more frequently observed in people over 65 years of age. The most common cause is left heart disease, followed by chronic obstructive pulmonary disease. In some regions, such as the United Kingdom, its prevalence has doubled over the past decade ^1^.

**Figure 1:**
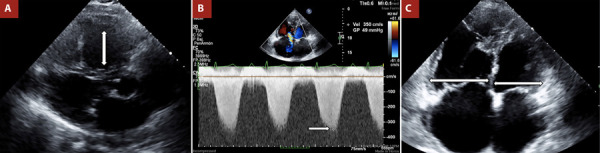
Echocardiographic findings of pulmonary hypertension.

Given its frequency and the high burden of morbidity and mortality, timely and accurate diagnosis of PH is essential, with echocardiography serving as the primary diagnostic tool. The main objective of echocardiography is to identify and characterize right heart pressure and volume overload. However, the geometry of the right ventricle (RV) is more complex and irregular compared to the left, making it difficult to assess RV function using a single parameter. Therefore, a multiparametric approach provides a more accurate characterisation of RV dysfunction and/or overload ^2^. By evaluating the morphology and function of both ventricles and atria, echocardiography allows the estimation of hemodynamic parameters and contributes to the identification of the underlying cause of PH, particularly when it is of intracardiac origin. Based on this information, the echocardiographic probability of PH can be estimated. The key echocardiographic parameter for this purpose is the tricuspid regurgitation velocity (TRV), which is used to estimate pulmonary artery systolic pressure, both serving as surrogates for direct measurements obtained by right heart catheterization ^3^ (Figure 1).

A. Parasternal long axis view showing right ventricular (RV) dilation in the outflow tract. B. Continuous wave Doppler of the tricuspid valve showing a regurgitation velocity >3.4 m/s. C. Apical four-chamber view with RV:LV ratio >1.

**Figure 2:**
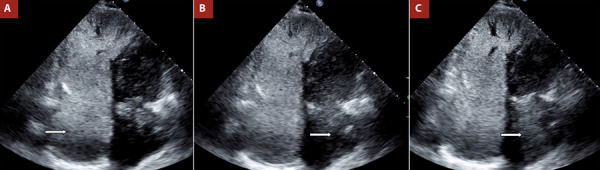
Contrast echocardiography with agitated saline: apical four-chamber view focused on the interatrial septum.

After excluding the two most common causes of PH discussed above, congenital heart disease becomes a major consideration, and echocardiography plays a central role at this stage of the diagnostic algorithm. In this context, the bubble test, or contrast echocardiography with agitated saline, has its greatest utility, either by detecting right-to-left (R-L) shunts or by enhancing the spectral Doppler signal to optimise measurements of tricuspid and pulmonary flow velocities ^4^ (Figure 2).

A.Initial injection: microbubbles in the right atrium (RA). B. Passage of microbubbles into the left atrium (LA) from the first cardiac cycle. C. Greater microbubble passage than in panel B. To view the complete test, see Video 1.

### Bubble test

The acoustic properties of bubbles enhance image quality by improving the delineation of cardiac structures, endocardial borders, and flow velocities. Macro-bubbles, or air bubbles larger than 10 μm in diameter, do not pass through the pulmonary capillaries, unlike microbubbles, which typically measure 2-4 μm. 

As the bubbles oscillate, the waves transmitting them change rapidly, along with their shape, which becomes asymmetrical, at varying frequencies and insonation angles. This produces vibrations and microcavitations that are primarily responsible for the enhanced echogenicity of the image ^5^. Using various techniques, such as harmonic imaging and Doppler modalities, artifacts can be reduced and image quality improved (including pulse inversion Doppler, power modulation, and contrast pulse sequencing) ^6^.

### Preprocedural considerations

Proper aseptic technique at the venous puncture site, a thorough understanding of relevant anatomy (e.g., injection should be performed in both arms if a persistent left superior vena cava or unroofed coronary sinus is suspected), clear explanation of the procedure, and informed consent are all essential components of the protocol. The procedure requires a venipuncture kit, a three-way stopcock, 10 or 20 mL of 0.9% saline solution (normal saline), and precise coordination of infusion timing with image acquisition, along with optimal visualization of the structures of interest, typically using a zoomed apical or subxiphoid view with focus on the interatrial septum.

### Indications

In which patients with PH should the bubble test/contrast echocardiography with agitated saline be performed?

As mentioned earlier, this test is particularly useful for detecting R-L shunts that may be a potential cause of PH. Additionally, it can enhance the spectral Doppler signal for the TRV jet and improve velocity measurements in the pulmonary artery ^7^.

Some clinical features that should raise suspicion of a shunt, and suggest potential benefit from performing a bubble test, include: atrial septal aneurysm (ASA) ^8^, right heart chamber dilation that is disproportionate to the severity of PH, paradoxical embolism, most notably in the context of a prior cryptogenic stroke (CS) ^9^, particularly when associated with deep vein thrombosis (DVT); and refractory hypoxemia, especially in acutely ill patients ^10^. Moreover, if more common causes of PH have been excluded and etiology remains unclear, the bubble test should be considered in all such patients ^11^.

A reasonable question in this context is whether the presence of PH increases the detection rates of intracardiac shunts, particularly patent foramen ovale (PFO) ^12^, given that it is a physiological opening and that the elevated R-L pressure gradient in PH could amplify the number of detectable cases or even confer a prognostic benefit by functioning similarly to an atrial septostomy. However, current evidence shows that PFO prevalence in patients with PH is comparable to that in the general population, and its presence does not appear to significantly influence prognosis ^13^.

### Safety considerations

Although there are no absolute contraindications to the bubble test, potential complications do exist, and certain patient groups have been less extensively studied. The most significant risk, given the injection of air into the venous circulation (with a recommended maximum of 1 mL), is the possibility of paradoxical embolism leading to ischemic stroke ^14^. Fortunately, this complication is rare, with a reported incidence of up to 0.15% of cases. When it does occur, it typically presents with mild and transient symptoms, and brain imaging has shown these to be small infarcts. No deaths related to the procedure have been reported. In pregnant and pediatric patients, current evidence is limited; however, small studies have shown no greater risk compared to the general population ^15^.

### Test protocol

- Provide a thorough explanation of the procedure and obtain informed consent.
- Position the patient lying on the echocardiography examination table.
- Prepare 8 mL of normal saline (0.9% NaCl), 1 mL of optional blood, 1 mL of air, a three-way stopcock, a venipuncture set (ideally 18G to allow adequate passage of bubbles), and two 10 mL syringes ^16^.
- One port of the three-way stopcock should be connected to the patient’s vein, typically the antecubital vein, although other options such as the femoral vein have been described. The other two ports are connected to the syringes: one containing the saline mixed with 1 mL of blood and 1 mL of air, and the second left empty. The port to the patient’s vein should be temporarily closed.
- After locating the appropriate echocardiographic window, vigorously agitate the saline solution for at least 10 to 20 passes over one minute between the two syringes, until a dense, white froth of bubbles is clearly visible.
- Open the stopcock port to the patient’s vein and inject the agitated saline at approximately 1 mL per second.
- As previously described, in transthoracic echocardiography (TTE), the preferred imaging views are the apical four-chamber view with zoom on the interatrial septum or the subxiphoid view. In transesophageal echocardiography (TEE), mid-esophageal views, short axis of the great vessels or bicaval view between 30° and 100°, are recommended. A recording of at least 10 cardiac cycles or 20 seconds is advised ^17^.

### Interventions to improve test performance

Multiple variables, including hemodynamic, technical, and preprocedural factors, can influence the quality and diagnostic yield of the bubble test. In cases where clinical uncertainty remains, several interventions can help optimise its performance:

- Add blood to the mixture: a composition of 10% blood, 10% air, and 80% normal saline (NS) produces smaller, denser microbubbles.
- Coughing, Valsalva maneuver, or abdominal compression: when performed during full opacification of the right atrium (RA), these actions transiently increase the R-L atrial pressure gradient.
- Valsalva maneuver: this should be performed once the bubbles have fully opacified the right-sided chambers. The patient is instructed to take a normal or deep inspiration followed by a forced expiration against a closed airway (with activation of the respiratory muscles), and to hold it for 15 to 20 seconds. This ensures adequate right atrial filling with bubbles. One easy way to confirm that the maneuver was effective is the observation of leftward bowing of the interatrial septum.
- Femoral vein injection: compared to antecubital access, infusion through the femoral vein is more likely to direct flow from the inferior vena cava toward the interatrial septum, whereas flow from the superior vena cava tends to be directed toward the tricuspid valve.
- Other described techniques include deep inspiration, forced expiration, or tilting of the examination table ^18^.

### Possible test outcomes

**Figure 3:**
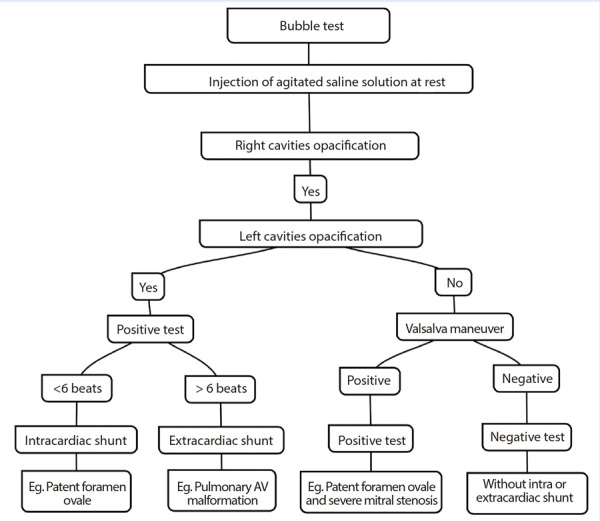
Contrast echocardiography with agitated saline - diagnostic algorithm.

The test is considered positive when microbubbles are observed passing into the left atrium (LA) (Figure 2 and Video 1). Semiquantification may be performed by counting bubbles in the frame where they are most clearly visible: mild (<10 microbubbles), moderate (10-30), and severe (>30), which helps estimate the magnitude of the shunt. The timing of microbubble appearance (before or after the fifth cardiac cycle) can also help determine the origin of the shunt, early passage (before the fifth beat) suggests an intracardiac source, while late passage indicates an extracardiac origin ^19^. Exceptions may occur in conditions of high cardiac output or in the presence of large pulmonary arteriovenous malformations (PAVMs), particularly when they drain directly into the inferior pulmonary veins; however, this remains an area of debate. At this stage, proper performance of the Valsalva maneuver is essential, as the leftward shift of the interatrial septum confirms its effectiveness. Figure 3 presents a proposed diagnostic algorithm incorporating the bubble test. It is important to note that the thresholds used to define positivity, both in terms of shunt magnitude and timing, are based primarily on case series and expert reports rather than clinical trials, but they are still widely accepted in current practice.

### False positives

Prolonged Valsalva maneuver, pseudocontrast in the LA, and a prominent Eustachian valve are potential sources of false-positive results. Possible solutions include repeating the Valsalva maneuver without contrast and using femoral vein access.

### False negatives

Inadequate contrast injection, improper performance of the Valsalva maneuver, a prominent Eustachian valve, and clinical conditions associated with elevated left-sided heart pressures, such as severe mitral valve disease, are potential causes of false-negative results ^20^.

### Negative contrast effect

This is an uncommon finding. It appears as a thin jet of clearing along the right edge of the interatrial septum, indicating a left-to-right shunt. However, the sensitivity of this finding is low.

### Other results

The expected and most common finding in the presence of a shunt is the passage of microbubbles from the RA to the LA. Less frequently, bubbles may be seen crossing in the opposite direction or simultaneously in both directions.

Some less common types of shunts can produce this pattern on the bubble test. For instance, when microbubbles appear in the LA before or simultaneously with their appearance in the RA, this suggests a sinus venosus (SV)-type defect or a persistent left superior vena cava (LSVC), and far less commonly, a shunt from the inferior vena cava (IVC) to the LA. In the first two cases, if bubbles are seen when contrast is injected from the lower limbs, an inferior SV-type defect should be suspected; conversely, if observed with upper limb injection, a superior SV-type defect is more likely. In the case of a persistent LSVC, bubbles appear only when the injection is administered through the left upper extremity. Shunts from the IVC to the LA may be either congenital or occur after surgical correction of an atrial septal defect (ASD), and in these cases, bubbles also appear first in the LA when injected from the lower limbs.

Other possible findings include the passage of microbubbles into the left ventricle (LV) followed by the RV in cases of ventricular septal defect (VSD) associated with PH, and into the descending aorta after the RV in cases of patent ductus arteriosus (PDA) with PH, both occurring before appearance in the LA ^21^.

### Perspectives

The temporal patterns of bubble appearance vary depending on the origin of the shunt. Although these differences may be imperceptible to the human eye, some studies using artificial intelligence (AI) algorithms have analysed acoustic intensity patterns to help identify the likely source of the shunt (22). This technology may enhance the diagnostic performance of contrast echocardiography and, in the future, could be applied in other areas, such as assisting residents in echocardiography training ^23^.

### Clinical case example

A 63-year-old woman with no significant cardiovascular history presented to the emergency department with chest pain. Electrocardiogram findings were unremarkable, and cardiac troponins were negative. An initial diagnosis of acute myocardial infarction was considered, and a transthoracic echocardiogram was performed. Left ventricular function was normal; however, the patient showed a high probability of PH (Figure 1). Notable findings included an ASA and right heart chamber dilation. As part of the workup for PH, a bubble test was performed and yielded a positive result (Figure 2 and Video 1). An ostium secundum-type ASD was confirmed, and percutaneous closure was indicated. The patient showed favorable clinical evolution during follow-up.

## Conclusions

The agitated saline or bubble test is a valuable tool in contrast echocardiography. It is accessible, cost-effective, safe, and easy to perform. This technique is particularly useful for the evaluation of intra- and extracardiac shunts, and in the context of PH, it can be a key diagnostic element when such shunts are suspected as the underlying cause. Clinical features that raise suspicion include right ventricular dysfunction that is disproportionate to the degree of PH, ASA, CS, and refractory hypoxemia, among others.
